# Short-term outcomes associated with fluoxetine, low-frequency rTMS, and their combination in first-onset adolescent OCD: a single-center retrospective cohort

**DOI:** 10.3389/fpsyt.2025.1663611

**Published:** 2025-11-05

**Authors:** Zhenzhen Yang, Chunfeng Hu, Huan Wang, Tao Yang, Haixia Wang, Meng Jiang

**Affiliations:** Department of Child and Adolescent Psychology, Daizhuang Hospital, Jining, Shandong, China

**Keywords:** adolescents, obsessive-compulsive disorder (OCD), fluoxetine, low-frequency repetitive transcranial magnetic stimulation (LF-rTMS), combined treatment

## Abstract

**Objective:**

This study aimed to compare the short-term clinical outcomes of low-frequency repetitive transcranial magnetic stimulation (LF-rTMS), fluoxetine, and their combination in adolescents with first-onset obsessive-compulsive disorder (OCD) using a single-center retrospective, non-randomized design.

**Methods:**

A single-center retrospective, non-randomized analysis was conducted on 167 adolescents (aged 12–18 years) diagnosed with obsessive-compulsive disorder (OCD) and treated at Dazhuang Hospital, Shandong Province, between January 2018 and June 2024. Based on treatment received, patients were categorized into three observational groups: LF-rTMS alone (n=32), fluoxetine alone (n=55), and combined fluoxetine plus LF-rTMS (n=80). LF-rTMS was delivered at 1 Hz over the right supplementary motor area (SMA), 20 sessions in total. Clinical outcomes were assessed using the Yale-Brown Obsessive-Compulsive Scale (Y-BOCS), Clinical Global Impressions-Improvement (CGI-I), Wisconsin Card Sorting Test (WCST), and quantitative electroencephalography (qEEG) at baseline, 2 weeks, and 4 weeks. Adverse events were monitored with the Treatment Emergent Symptom Scale (TESS).

**Results:**

After 4 weeks of treatment, 68.26% of patients overall met the predefined response threshold (≥35% Y-BOCS reduction), with rates of 34.38% in the LF-rTMS group, 63.64% in the fluoxetine group, and 85.00% in the combined treatment group; remission (Y-BOCS ≤12) was observed in 12.5%, 20.0%, and 32.5% of the groups, respectively (χ²=27.85, P<0.001). Repeated-measures analyses further indicated significant Time and Time×Group effects for both Y-BOCS and CGI-I scores (P<0.001), confirming differential symptom trajectories across treatment groups. The combined group also showed comparatively greater improvements in cognitive performance (WCST indices) and more favorable qEEG changes relative to the monotherapy groups (P<0.001). No statistically significant differences in the distribution of adverse reactions were observed among the three groups (P = 0.549).

**Conclusion:**

The findings suggest that combining fluoxetine with LF-rTMS may be associated with greater short-term improvements in symptom severity, cognitive function, and neural activity compared with monotherapy, while maintaining a favorable safety profile.

## 1 Background

Obsessive-Compulsive Disorder (OCD) is a prevalent chronic mental disorder marked by recurrent obsessive thoughts and compulsive behaviors, significantly affecting patients’ daily lives and mental well-being (1). Although OCD can manifest at any age, it commonly emerges during childhood, adolescence, or early adulthood. Research indicates that early adulthood, particularly between the ages of 19 and 30, represents a key peak for OCD onset, with a prevalence estimated between 1-3% (2). Furthermore, individuals aged 19–25 account for 14.1% of all OCD cases (3) but symptom progression tends to be slower compared to childhood or adolescence during this stage. In childhood and adolescence, the prevalence of OCD is estimated to be between 0.1% and 2.3% in the general population (4), accounting for approximately 48.4% of all OCD cases (3). Early-onset OCD during childhood or adolescence is more likely to go unrecognized or be misunderstood by parents and teachers, leading to delays in diagnosis and treatment, which can exacerbate the condition. Additionally, these stages are crucial for physical and psychological development. Due to their reliance on parental support, children and adolescents may face greater challenges in accessing professional mental health care when symptoms arise (5). Besides, improper treatment during these vulnerable years can increase the risk of developing other psychiatric disorders in adulthood (6).

As we know above, adolescence is a critical phase for both physical and psychological development; thus, the onset of OCD during this period can disrupt academic performance and social interactions while also having lasting implications for mental health. Therefore, early intervention and preventive strategies are essential when initial symptoms appear, as they may prevent OCD from progressing into a more severe and chronic condition (7, 8). Traditional treatment methods, including cognitive behavioral therapy (CBT) and pharmacotherapy, can alleviate symptoms and enhance patient functioning to some extent. However, given the complex nature of many mental health disorders, a combination approach is often necessary to address both neurochemical imbalances and maladaptive behavioral problem. The primary treatment approach has shifted toward pharmacotherapy combined with conventional behavioral therapy, utilizing common medications such as fluoxetine, sertraline, paroxetine, and clomipramine (9). Among these, fluoxetine—a selective serotonin reuptake inhibitor (SSRI)—works by selectively inhibiting the reuptake of serotonin (5-HT) in central neurons, increasing its concentration in the synaptic cleft (10). While fluoxetine is primarily used for various depressive disorders, it is also widely employed in treating OCD. Although existing studies have shown fluoxetine’s effectiveness for psychiatric disorders, the efficacy of traditional treatments combining pharmacotherapy with general behavioral therapy often remains limited. When patients do not experience substantial short-term benefits or encounter adverse reactions, their compliance may decline, leading to chronic or treatment-resistant OCD. Consequently, our focus has shifted toward integrating other effective adjunctive measures to enhance patients’ quality of life.

Low-Frequency Repetitive Transcranial Magnetic Stimulation (LF-rTMS) is a non-invasive neuromodulation technique that modulates neural activity in the cerebral cortex using low-frequency magnetic fields. Recognized as an effective treatment with Grade A evidence in accordance with established guidelines, such as the *Clinical Practice Guidelines for the Therapeutic Use of Repetitive Transcranial Magnetic Stimulation in Neuropsychiatric Disorders*, low-frequency repetitive transcranial magnetic stimulation (LF-rTMS) has demonstrated significant benefits in the treatment of conditions like depression and anxiety (11). Furthermore, numerous studies indicate that LF-rTMS is the most effective strategy among all rTMS approaches for reducing neural activity in the dorsolateral prefrontal cortex of OCD patients (12–16). In treating first-onset OCD, a clinical approach has emerged that utilizes LF-rTMS alone, as these patients often demonstrate a favorable prognosis with active treatment, and positive outcomes have been documented (17–19). Despite this, there is currently a lack of systematic and comprehensive research examining the combined efficacy of fluoxetine and LF-rTMS in first-onset adolescent OCD patients. It remains uncertain whether the effects of LF-rTMS alone differ from those when it is used in conjunction with fluoxetine. This study aims to conduct a retrospective analysis to explore the therapeutic effects and adverse reactions associated with the combination of fluoxetine and LF-rTMS in adolescent OCD patients. Our goal is to provide new insights and methods for treating adolescent OCD, contributing scientific evidence to support its application in clinical practice.

## 2 Methods

### 2.1 Study design and subjects

This was a single-center, retrospective observational study that collected clinical data from patients visiting the psychiatric outpatient department at Dazhuang Hospital, Shandong Province, between January 1, 2018, and June 30, 2024. Patients were allocated into three treatment groups—LF-rTMS alone (n=32), fluoxetine alone (n=55), and combined treatment (n=80)—based on post-admission treatment modalities determined by patient/caregiver preference, clinical suitability, and treatment accessibility, rather than randomization. Consequently, baseline differences—including unmeasured factors such as motivation, insight, or family support—may have influenced outcomes, introducing selection bias and precluding causal inference from between-group comparisons. For this reason, all analyses are presented as associations rather than causal effects. The study received approval from the Ethics Committee of Dai Zhuang Hospital, Shandong Province, with the ethics approval number: 2022 Scientific Research No. 05 - 202201KS-1, and all procedures adhered to the Declaration of Helsinki.

Subjects meeting the following criteria were included:

Inclusion Criteria:

(1) Diagnosed by two psychiatrists as meeting DSM criteria (20) for OCD, characterized by recurrent obsessions and/or compulsions that cause significant anxiety or distress and severely impact daily life, occupational functioning, or social activities. (2) Yale-Brown Obsessive-Compulsive Scale (Y-BOCS) (21) score of ≥16. (3) Age between 12 and 18 years. (4) No contraindications to LF-rTMS treatment. (5) Determined by psychiatrists to be suitable for treatment with fluoxetine and LF-rTMS. (6) No psychiatric medication treatment in the last month.

Exclusion Criteria:

(1) Contraindications to LF-rTMS, including implants, a history of epilepsy, severe heart disease or recent cardiac events, and history of brain trauma or tumors. (2) Use of psychiatric or hormonal medications in the last month. (3) Severe somatic or neurological diseases. (4) Presence of other severe psychiatric disorders. (5) High risk of suicide. (6) History of drug abuse or alcohol dependence. (7) Underwent electroconvulsive therapy, LF-rTMS, or transcranial direct current stimulation in the past three months.

Finally, a total of 32, 55 and 80 patients were included in the LF-rTMS alone, fluoxetine alone and combined treatment groups, respectively.

### 2.2 Interventions

(1) LF-rTMS Group: This group exclusively received LF-rTMS therapy (22), administered by professionally trained technicians to ensure standardization and minimize bias. LF-rTMS was delivered with a figure-of-eight coil (Nerosoft™, Russia) targeting the right supplementary motor area (SMA). The resting motor threshold (RMT) was determined over the left primary motor cortex (M1) hand area by recording motor-evoked potentials (MEPs) from the contralateral thenar muscle; RMT was defined as the minimum intensity that elicited MEPs ≥50 μV in at least 5 of 10 consecutive trials. Stimulation over the SMA was then applied at 1 Hz, 100% of the individual M1-derived RMT, for 20 minutes (1,200 pulses) per session, four sessions per week for five weeks (20 sessions in total). SMA targeting was achieved by MRI-guided neuronavigation to the medial frontal cortex (Brodmann area 6) when available; otherwise, the scalp target was localized using the 10–20 system at a point ~15% of the nasion–inion distance anterior to Cz on the midline, then shifted 1–2 cm to the right to approximate the right SMA. The coil handle was oriented posterior-laterally, and stimulation parameters were kept constant across sessions. This intensity-referencing and targeting approach follows prior rTMS protocols in OCD.
(2) Fluoxetine Group: All patients in this group received fluoxetine (20 mg × 14 capsules, approval number: H19980114, Shanghai Zhongxi Pharmaceuticals Co., Ltd.). The initial daily dose was 20 mg, which was increased to 40 mg after two weeks to effectively manage psychiatric symptoms without significant side effects. This treatment lasted for four weeks.
(3) Combined Treatment Group: Patients in this group received both fluoxetine and LF-rTMS treatments, following the procedures outlined for the LF-rTMS Group and the Fluoxetine Group.

Resulted in three groups of patients comprising 32, 55, and 80 cases, respectively.

### 2.3 General information and assessment metrics

In this retrospective study, we extracted various general information from electronic medical records, including age, years of education, gender, body mass index, and type of OCD for patients in the three groups. Additionally, we retrieved scoring data for all patients before treatment, as well as at 2 and 4 weeks post-treatment. The assessment metrics included the following:

Yale-Brown Obsessive-Compulsive Scale (Y-BOCS) scores: This scale consists of 10 items used to measure the severity of obsessive thoughts and compulsive behaviors in OCD. Each item is scored from 0 (no symptoms) to 4 (severe symptoms), yielding a total score range of 0 to 40, where higher scores indicate more severe symptoms. For details, see **Supplementary materials 1**.

Treatment efficacy rate: A reduction in the Y-BOCS score of ≥35% from baseline is defined as effective treatment, while a reduction of <35% is classified as ineffective.

Treatment Emergent Symptom Scale (TESS): This scale recorded adverse reactions observed in the three groups during treatment, including symptoms such as spasms, hearing loss, tinnitus, syncope, local neck pain, headache, and subjective discomfort. For details, see **Supplementary materials 2**.

Clinical Global Impressions-Improvement (CGI-I): The CGI-I scale evaluated treatment outcomes, with lower scores indicating better clinical results. For details, see **Supplementary materials 3**.

Wisconsin Card Sorting Test (WCST): Cognitive performance was assessed using the WCST, which includes metrics such as the total number of trials administered, number of correct responses, number of random errors, number of perseverative errors, and the ability to complete classification tasks.

Quantitative Electroencephalography (qEEG): Resting-state EEG was acquired in a quiet, dimly lit room with participants seated comfortably and instructed to keep their eyes closed and to minimize movement while a technician continuously monitored vigilance and motion. Signals were recorded with a digital electroencephalograph (23) using the international 10–20 system; electrode impedance was maintained at ≤5 kΩ. The sampling frequency was 200 Hz, the time constant 0.3 s, and a band-pass filter of 1–35 Hz was applied with a 50-Hz notch filter for line noise as needed. Sixteen recording electrodes were used for spectral analysis, corresponding to the frontal region (F: F3+F4+F7+F8+Fx), central region (C: C3+C4+Cx), lateral temporal region (T: T3+T4+T5+T6), medial parietal region (P: P3+P4+Pz), and occipital region (O: O1+O2). Recordings employed a linked-earlobe reference with a monopolar montage, and each subject underwent approximately 30 minutes of EEG recording. Data were visually inspected and preprocessed in Nihon Kohden QP-160AK. Ocular artifacts (blinks/saccades), muscle activity, and gross movement artifacts were identified using combined visual review and semi-automatic thresholds; epochs were rejected if peak-to-peak amplitude exceeded ~100 μV in any channel, if EOG/frontal deflections exceeded ~ ± 75 μV, if high-frequency muscular contamination (≈30–80 Hz) was present, or if flat-line/step artifacts were detected. The continuous data were segmented into non-overlapping 2-s epochs, and only artifact-free epochs were retained. A minimum of ≥180 s artifact-free data per assessment was required for inclusion in spectral analyses; recordings not meeting this criterion were excluded from qEEG analyses. Drowsiness-related segments (e.g., α attenuation with diffuse θ bursts) were identified and excluded. Power spectral density was estimated with Welch’s method (Hanning window, 2-s window length, 50% overlap), and absolute power was computed for δ (0.5–4 Hz), θ (4–8 Hz), α (8–13 Hz), and β (13–30 Hz) bands. Regional absolute power values were calculated by averaging electrodes within each ROI (F, C, T, P, O) as defined above. To stabilize variance, absolute power values were log10-transformed prior to statistical modeling. This standardized acquisition and preprocessing pipeline was implemented to maximize precision and reproducibility while minimizing the influence of non-neural artifacts.

### 2.4 Statistical methods

Normally distributed continuous data were expressed as mean ± standard deviation (Mean ± SD) and compared between groups using one-way ANOVA. For non-normally distributed continuous data, values were expressed as median and interquartile range [M (Q1, Q3)] and analyzed using the Kruskal-Wallis H test. Categorical data were presented as counts and percentages [n (%)] and compared using the Pearson χ² test or Fisher’s exact test. The Standardized Mean Difference (SMD) was also employed to assess intergroup differences: SMD < 0.10 indicates acceptable balance between groups, 0.10-0.34 indicates small differences, 0.35-0.64 indicates moderate differences, 0.65-1.19 indicates large differences, and SMD ≥ 1.20 indicates very large differences (24). For repeated-measures outcomes, linear mixed-effects models (LMMs) were used for Y-BOCS scores, with fixed effects for Time (baseline, week 2, week 4), Group (LF-rTMS, fluoxetine, combination), and their interaction, and a random intercept for participants. For CGI-SI scores, generalized estimating equations (GEE) with an exchangeable correlation structure were applied. Omnibus significance was assessed using likelihood ratio tests (LRT) for LMMs and Wald chi-square tests for GEEs, with model-estimated marginal means (EMM ± SE) reported. For WCST scores and qEEG metrics, repeated-measures analyses were also conducted using LMM or GEE as appropriate, and estimated marginal means were reported. Categorical clinical efficacy rates and adverse reactions were analyzed using χ² or Fisher’s exact test. P < 0.05 (two-tailed) was considered statistically significant.

## 3 Results

### 3.1 Comparison of baseline characteristics among three groups

A total of 167 participants were included: 32 in the LF−rTMS group (19.16%), 55 in the fluoxetine group (32.93%), and 80 in the combined treatment group (47.90%). Baseline characteristics were broadly comparable across groups, with no statistically significant differences in age, years of education, illness duration, baseline Y−BOCS scores, OCD symptom subtype distribution, family psychiatric history, or socioeconomic status (all P>0.05; SMDs ≤0.23). For details, see **Table 1**.

**Table 1:** Baseline characteristics of the three patient groups.

Variables	LF-rTMSz group (*n* = 32)	Fluoxetine group (*n* = 55)	Combined treatment group(*n* = 80)	P value	SMD
Age, Mean ± SD	14.594 ± 1.775	15.200 ± 1.919	14.988 ± 1.852	0.344	0.219
Education, Mean ± SD	8.656 ± 1.961	9.164 ± 1.844	9.025 ± 1.807	0.462	0.179
Illness duration (months), Mean ± SD	9.188 ± 2.838	9.129 ± 2.961	9.228 ± 2.773	0.981	0.015
Types, *n* (%)				0.942	0.227
Compulsive emotions	8 (25.000)	12 (21.818)	22 (27.500)		
Compulsive behavior	7 (21.875)	8 (14.545)	13 (16.250)		
Obsessive thoughts	5 (15.625)	12 (21.818)	17 (21.250)		
Compulsive intention	4 (12.500)	8 (14.545)	7 (8.750)		
Multiplicity	8 (25.000)	15 (27.273)	21 (26.250)		
Family psychiatric history, n (%)				0.299	0.12
Yes	7 (21.88)	13 (23.64)	27 (33.75)		
No	25 (78.12)	42 (76.36)	53 (66.25)		
Socioeconomic status, n (%)				0.775	0.073
Low	12 (37.50)	24 (43.64)	26 (32.50)		
Middle	16 (50.00)	24 (43.64)	42 (52.50)		
High	4 (12.50)	7 (12.73)	12 (15.00)		
Y-BOCS scores, Mean ± SD	21.438 ± 2.711	21.255 ± 2.647	21.700 ± 2.978	0.66	0.106

### 3.2 Comparison of efficacy and adverse reactions among the three groups

In the Fluoxetine group, after 4 weeks of treatment, 36.36% of participants were ineffective, while 63.64% were effective. In the LF-rTMS group, 65.62% were ineffective, and 34.38% were effective. The combined treatment group had 15.00% ineffective participants and 85.00% effective. A statistically significant difference in efficacy rates was observed among the three groups after 4 weeks of treatment (χ²=27.85, P<0.001). Additionally, no statistically significant difference was found in the distribution of adverse reactions among the three groups (Fisher’s exact test, P = 0.549). For details, see **Table 2**.

**Table 2:** Comparison of efficacy and adverse reactions between three groups.

Variables	Total	LF-rTMSz group(n = 32)	Fluoxetine group(n = 55)	Combined treatment group(n = 80)	Statistic	P
Total clinical efficacy, n(%)					χ²=27.85	<.001
Ineffective	53 (31.74)	21 (65.62)	20 (36.36)	12 (15.00)		
Effective	114 (68.26)	11 (34.38)	35 (63.64)	68 (85.00)		
Adverse reactions, n(%)					Fisher exact	0.549
Nausea	8 (4.79)	1 (3.12)	2 (3.64)	5 (6.25)		
Elevation of blood pressure	6 (3.59)	1 (3.12)	1 (1.82)	4 (5.00)		
Drowsiness	16 (9.58)	3 (9.38)	5 (9.09)	8 (10.00)		
Dizziness	6 (3.59)	2 (6.25)	0 (0.00)	4 (5.00)		
Others	7 (4.19)	2 (6.25)	4 (7.27)	1 (1.25)		
Nothing	124 (74.25)	23 (71.88)	43 (78.18)	58 (72.50)		

### 3.3 Comparison of Y-BOCS and CGI-SI scores before and after treatment in the three groups

In the present study, a comprehensive analysis was conducted on the Y-BOCS and the CGI-SI scores across different treatment groups before and after the intervention (see **Table 3**). Significant statistical differences were observed. Notably, after two weeks of treatment, there was a significant reduction in Y-BOCS scores across all groups compared to their baseline scores (F = 9.39, P<0.001), and the CGI-SI scores also showed marked improvements (F = 34.87, P<0.001). These findings indicate an appreciable short-term effect of the treatments administered. By the fourth week, the initial improvements were not only sustained but also enhanced. The Y-BOCS scores saw further reductions (F = 11.31, P<0.001), and CGI-SI scores continued to improve (F = 36.10, P<0.001). When comparing results from the second week to the fourth week of treatment, significant differences in Y-BOCS scores (∮p<0.001) and CGI-SI scores (∮p<0.001) were noted among the three groups, evidencing a continuous trend of improvement and underscoring the benefit of extending treatment duration to enhance therapeutic outcomes. Moreover, throughout different stages of treatment, the combination therapy group consistently exhibited significantly lower Y-BOCS and CGI-SI scores than both the LF-rTMS group and the fluoxetine group (P<0.001). In addition, repeated-measures analyses were conducted to account for within-subject correlations. LMMs for Y-BOCS and GEEs for CGI-SI confirmed significant main effects of Time and significant Time×Group interactions (all P<0.001), indicating that symptom improvement trajectories differed across treatment groups. Estimated marginal means (EMM ± SE) from these models are presented in **Table 4**, showing that the combination group achieved the greatest reductions by week 4. Omnibus test results are summarized in **Table 5**, further supporting that both Y-BOCS and CGI-SI exhibited significant overall improvements over time, with the combination therapy producing more favorable short-term outcomes than monotherapies.

**Table 3:** Comparison of Y-BOCS and CGI-SI scores before and after treatment in three groups of patients.

Group	Case	Baseline	Treatment for two weeks	Treatment for four weeks	F	P value
Y-BOCS						
LF-rTMSz group	32	21.44 ± 2.71	20.06 ± 2.73*	18.88 ± 3.00*	6.38	0.003
Fluoxetine group	55	21.25 ± 2.65	19.60 ± 2.83*	17.76 ± 3.05*	9.67	<0.001
Combined treatment group	80	21.70 ± 2.98	17.66 ± 3.54*#†	15.72 ± 3.91*#†	21.57	<0.001
F		0.42	9.39	11.31		
P value		0.66	<.001	<.001		
CGI-SI						
LF-rTMSz group	32	4.97 ± 0.65	3.97 ± 0.59*	3.12 ± 0.91*∮	115.89	<0.001
Fluoxetine group	55	5.00 ± 0.61	3.91 ± 0.80*	2.27 ± 0.78*∮	114.15	<0.001
Combined treatment group	80	5.00 ± 0.55	3.09 ± 0.56*#†	1.59 ± 0.94*#†∮	47.96	<0.001
F		0.04	34.87	36.10		
P value		0.964	<.001	<.001		

Statistical significance was set at p<0.05.

*p<0.001, compared with the baseline group.

∮p<0.001, compared with the two-week treatment group.

#p<0.001, compared with the LF-rTMS group.

†p<0.001, compared with the combined treatment group.

**Table 4:** Estimated marginal means (EMM ± SE) of Y-BOCS and CGI-SI scores from repeated-measures models (LMM/GEE) at baseline, week 2, and week 4 in each treatment group.

Group	Time	EMM ± SE	
		Y-BOCS	CGI-SI
LF-rTMSz group	baseline	21.44 ± 0.56	4.97 ± 0.13
	week2	20.06 ± 0.56	3.97 ± 0.13
	week4	18.88 ± 0.56	3.13 ± 0.13
Fluoxetine group	baseline	21.25 ± 0.43	5.0 ± 0.10
	week2	19.6 ± 0.43	3.91 ± 0.10
	week4	17.76 ± 0.43	2.27 ± 0.10
Combined treatment group	baseline	21.7 ± 0.35	5.0 ± 0.08
	week2	17.66 ± 0.35	3.09 ± 0.08
	week4	15.73 ± 0.35	1.59 ± 0.08

Values are estimated marginal means (EMM) ± standard error (SE) derived from repeated-measures analyses. Y-BOCS scores were analyzed using a linear mixed-effects model (LMM), and CGI-SI scores using generalized estimating equations (GEE). Fixed effects included Time (baseline, week 2, week 4), Group (LF-rTMS, fluoxetine, combination), and their interaction, with a random intercept for participants. Lower scores indicate lower symptom severity.

**Table 5:** Omnibus tests of main and interaction effects for Y-BOCS and CGI-SI based on repeated-measures analyses (LMM/GEE).

Outcome	Effect	Test	Chi2	Df	P-value
Y-BOCS	Time	LRT (LMM)	412.12	2.00	<.01
	Group		10.84	2.00	0.01
	Time×Group		115.45	4.00	<.01
CGI-I	Time	Wald (GEE)	1451.33	2.00	<.01
	Group		0.07	2.00	0.97
	Time×Group		62.70	4.00	<.01

Results are from omnibus tests of repeated-measures analyses. For Y-BOCS, a linear mixed-effects model (LMM) was applied; omnibus significance was assessed using likelihood ratio tests (LRT). For CGI-SI, generalized estimating equations (GEE, exchangeable correlation structure) were used; omnibus significance was assessed using Wald chi-square tests. Reported values are chi-square statistics (χ²), degrees of freedom (df), and two-tailed P values. Significant Time effects indicate overall symptom change across baseline, week 2, and week 4. Significant Group and Time×Group interactions reflect differences in average levels and improvement trajectories among treatment groups.

### 3.4 Comparison of cognitive function scores between groups

**Figure 1:**
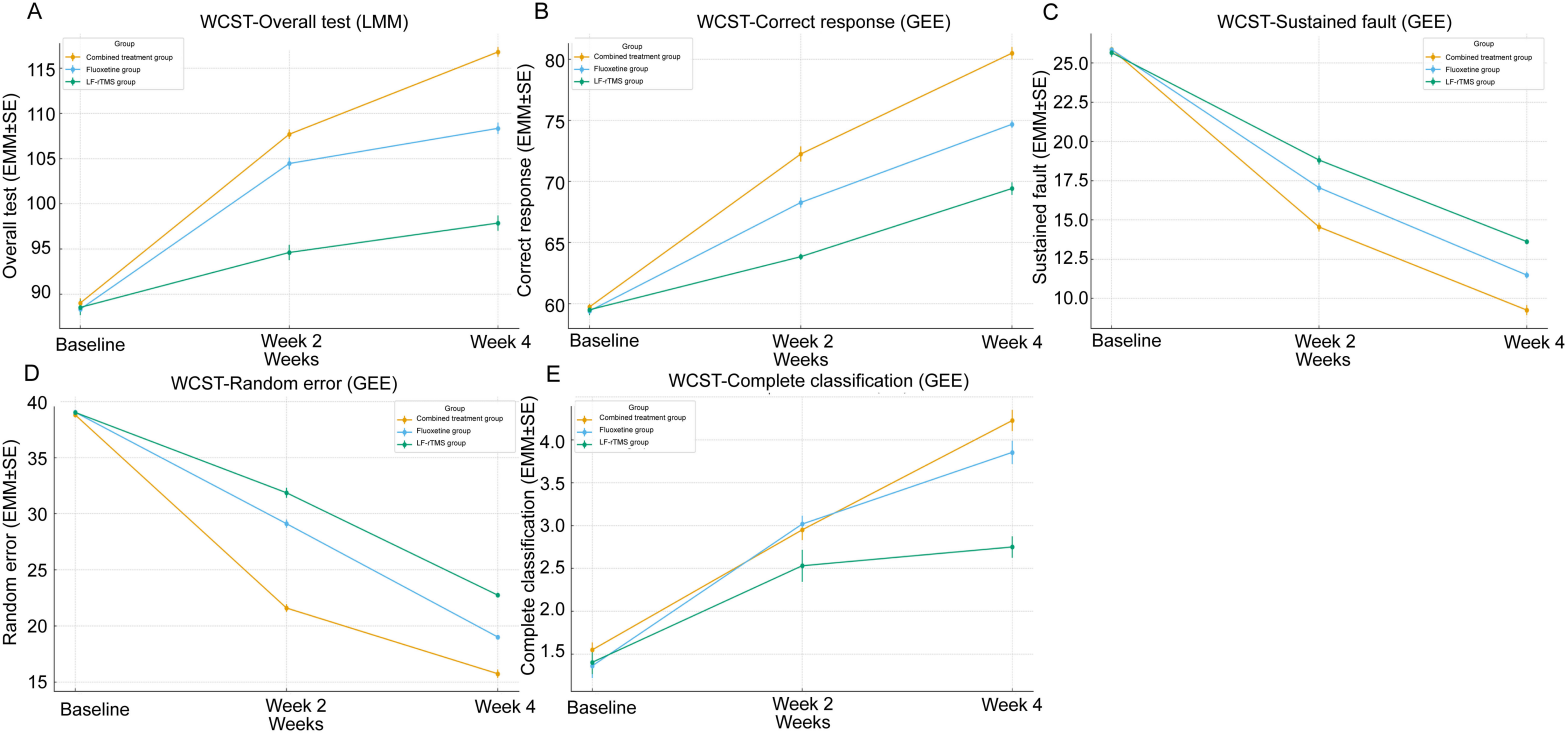
Model-estimated marginal means (EMM ± SE) of WCST performance over time in adolescents with OCD. **(A)** Overall test (LMM); **(B)** Correct responses (GEE); **(C)** Perseverative errors (GEE); **(D)** Random errors (GEE); **(E)** Complete classification (GEE).

As illustrated in **Figure 1**, a noticeable increase in Overall test, correct responses, and complete classification scores was observed across all three groups when compared to baseline levels before treatment. Specifically, by the end of week 2, all groups exhibited significant improvements, with further enhancements noted by week 4. Conversely, both the number of Perseverative errors and Random errors on the WCST showed a consistent decline from baseline through weeks 2 and 4, reflecting an overall reduction in cognitive errors as treatment progressed. Repeated-measures analyses (LMM for Overall test; GEE for other WCST indicators) confirmed significant main effects of Time (all P < 0.001) and Time×Group interactions (all P < 0.01), indicating that the trajectories of cognitive improvement differed between treatment groups. Estimated marginal means (EMM ± SE) demonstrated that, particularly by week 4, the combined treatment group achieved the most favorable outcomes, with higher scores in correct responses and complete classification, and lower scores in perseverative and random errors, compared with the LF-rTMS and fluoxetine groups.

### 3.5 Comparison of absolute power values in different brain regions across frequency bands among the three groups

After four weeks of treatment, qEEG analyses revealed notable changes across different brain regions among the three groups, particularly in the combined treatment group. In theαfrequency band (see **Table 6**), after four weeks of treatment, the combined treatment group exhibited a significant increase in absolute power values across all recorded brain regions compared to baseline levels. This increase was substantially higher than those observed in both the LF-rTMS group and the Fluoxetine group, with statistical significance noted (P<0.001). Specifically, improvements in the frontal (F), central (C), and occipital (O) regions were remarkable, underscoring enhanced neural activity post-treatment. In theβfrequency band (see **Table 7**), there were differential responses observed across brain regions. The combined treatment group demonstrated lower absolute power values in the frontal, central, and parietal regions compared to the LF-rTMS group (P<0.001), indicating a decrease in neural excitability in these areas. Conversely, in the occipital region, the combined group showed higher power values than both the LF-rTMS group and the Fluoxetine group (P<0.001), suggesting region-specific enhancements in activity. For the θ frequency band (see **Table 8**), the combined treatment group showed significant improvements in absolute power values compared to the other groups in all regions except the central region, where no significant differences were noted (P<0.001). This suggests a generalized enhancement of theta activity, which is often associated with relaxation and reduced anxiety levels. In the δ frequency band (see **Table 9**), the results were consistent with a pronounced reduction in absolute power values in all brain regions for the combined treatment group when compared to both the LF-rTMS group and the Fluoxetine group (P<0.001). This decrease could reflect deep relaxation states and potential therapeutic effects on subcortical areas implicated in mood regulation. Notably, no significant differences were observed between the combined treatment group and the Fluoxetine group in theβfrequency band across the frontal, central, and temporal regions, suggesting a similar effect of these treatments on these specific brain areas.

**Table 6:** Comparison of absolute power values in the α frequency band across brain regions.

Spectrum	Group	Time	F	C	T	P	O
α	LF-rTMSz group	Baseline	9.20 ± 1.06	6.47 ± 1.33	9.67 ± 1.42	14.73 ± 2.10	27.13 ± 2.97
		Treatment for four weeks	9.13 ± 1.17	7.30 ± 0.70*	10.50 ± 1.11*	14.57 ± 2.05	29.63 ± 2.50*
	Fluoxetine group	Baseline	9.40 ± 1.25	6.40 ± 1.10	10.17 ± 1.44	14.43 ± 2.39	26.87 ± 2.65
		Treatment for four weeks	9.70 ± 1.60	8.33 ± 1.71*	10.73 ± 1.53	14.77 ± 2.47	30.17 ± 3.15*
	Combined treatment group	Baseline	9.33 ± 1.79	6.70 ± 1.26	10.57 ± 1.28	15.27 ± 2.10	28.13 ± 2.71
		Treatment for four weeks	12.27 ± 1.60*#†	10.67 ± 1.45*#†	12.40 ± 1.94*#†	19.03 ± 2.54*#†	35.57 ± 3.01*#†

Statistical significance was set at p<0.05.

#p<0.001, compared with the LF-rTMS group.

†p<0.001, compared with Fluoxetine group.

**Table 7:** Comparison of absolute power values in the β frequency band across brain regions.

Spectrum	Group	Time	F	C	T	P	O
β	LF-rTMSz group	Baseline	7.67 ± 0.71	8.20 ± 0.85	7.10 ± 0.92	13.03 ± 0.81	5.47 ± 1.66
		Treatment for four weeks	7.07 ± 1.34*	7.63 ± 1.00*	6.23 ± 1.50*	12.10 ± 0.80*	7.30 ± 1.09*
	Fluoxetine group	Baseline	7.43 ± 0.86	8.00 ± 1.02	6.97 ± 1.07	12.77 ± 1.04	4.83 ± 1.90
		Treatment for four weeks	5.80 ± 1.45*	6.50 ± 1.46*	5.97 ± 1.25*	10.83 ± 0.79*	8.93 ± 0.87*
	Combined treatment group	Baseline	7.50 ± 0.68	7.93 ± 0.94	6.83 ± 0.59	12.73 ± 1.11	5.40 ± 1.69
		Treatment for four weeks	5.77 ± 1.41*#	6.00 ± 1.62*#	5.60 ± 1.04*	9.13 ± 1.38*#†	10.20 ± 1.30*#†

Statistical significance was set at p<0.05.

#p<0.001, compared with the LF-rTMS group.

†p<0.001, compared with Fluoxetine group.

**Table 8:** Comparison of absolute power values in the θ frequency band across brain regions.

Spectrum	Group	Time	F	C	T	P	O
θ	LF-rTMSz group	Baseline	9.37 ± 1.27	5.40 ± 1.07	10.67 ± 1.12	7.57 ± 1.10	13.20 ± 1.77
		Treatment for four weeks	8.07 ± 0.83*	4.43 ± 1.33*	9.60 ± 1.30*	6.97 ± 1.50	12.30 ± 1.82
	Fluoxetine group	Baseline	9.37 ± 1.19	5.67 ± 1.24	10.50 ± 1.01	7.17 ± 1.18	12.47 ± 2.36
		Treatment for four weeks	7.50 ± 2.22*	4.23 ± 1.48*	9.17 ± 1.37*	6.47 ± 1.43*	11.80 ± 1.13
	Combined treatment group	Baseline	9.33 ± 1.12	5.13 ± 1.25	10.57 ± 0.94	6.87 ± 1.20	12.90 ± 2.34
		Treatment for four weeks	4.83 ± 1.46*#†	4.10 ± 1.95*	7.27 ± 0.91*#†	4.30 ± 1.06*#†	8.73 ± 1.08*#†

Statistical significance was set at p<0.05.

#p<0.001, compared with the LF-rTMS group.

†p<0.001, compared with Fluoxetine group.

**Table 9:** Comparison of absolute power values in the δ frequency band across brain regions.

Spectrum	Group	Time	F	C	T	P	O
δ	LF-rTMSz group	Baseline	8.60 ± 1.07	6.53 ± 1.46	8.63 ± 1.22	6.00 ± 2.57	7.13 ± 1.74
		Treatment for four weeks	7.67 ± 1.67*	5.93 ± 1.64	7.03 ± 1.85*	5.90 ± 1.49	6.67 ± 1.30
	Fluoxetine group	Baseline	8.60 ± 1.22	6.60 ± 1.25	8.13 ± 1.22	6.40 ± 2.63	7.27 ± 1.46
		Treatment for four weeks	7.33 ± 1.12*	4.57 ± 1.41*	6.00 ± 1.70*	5.40 ± 1.38	6.70 ± 1.26
	Combined treatment group	Baseline	8.73 ± 1.20	6.17 ± 1.12	7.93 ± 1.28	6.90 ± 2.78	7.53 ± 1.38
		Treatment for four weeks	6.03 ± 1.50*#†	3.17 ± 1.51*#†	4.57 ± 0.97*#†	4.37 ± 1.43*#†	5.13 ± 1.38*#†

Statistical significance was set at p<0.05.

#p<0.001, compared with the LF-rTMS group.

†p<0.001, compared with Fluoxetine group.

## 4 Discussion

Research over the past few decades indicated that 1% to 4% of individuals worldwide—encompassing children, adolescents, and adults—will experience obsessive-compulsive disorder (OCD) at some point in their lives (25). OCD can begin in childhood and is often considered a neurodevelopmental phenomenon, commonly referred to as Early Onset OCD (1, 26). In recent years, particularly following the outbreak of the COVID-19 pandemic, there has been a notable increase in the incidence of Obsessive-Compulsive Disorder (OCD), especially among at-risk populations such as children, adolescents, pregnant women, and healthcare workers (27, 28). Moreover, over 50% of adults with OCD reported experiencing symptoms during childhood and adolescence, yet many did not receive adequate treatment (29). This gap may be attributed to imbalances in brain development during adolescence and environmental factors, including connectivity issues between the prefrontal cortex and limbic system (30), which can lead to impulse control disorders and compulsive behaviors. Without timely intervention, OCD can negatively impact academic performance, interpersonal relationships, and increase the risk of anxiety or depression. Prolonged stress may also contribute to dysfunction of the hypothalamic-pituitary-adrenal (HPA) axis, further affecting emotional regulation and learning (31). Treatment for adolescents with first-onset OCD typically involves a combination of pharmacotherapy, psychotherapy, and physical therapies (32). Selective serotonin reuptake inhibitors (SSRIs) like fluoxetine help alleviate symptoms by regulating neurotransmitter levels; however, they may also cause side effects that lead some patients to explore non-pharmacological therapies, such as cognitive-behavioral therapy (CBT) and repetitive transcranial magnetic stimulation (rTMS) (33, 34). These methods can assist in behavioral correction and cognitive adjustment, helping patients reduce the frequency of obsessive-compulsive symptoms.

Low-frequency repetitive transcranial magnetic stimulation (LF-rTMS) is a non-invasive neuromodulation technique that utilizes low-frequency stimulation (typically ≤1 Hz) to temporarily inhibit regional brain activity, while high-frequency stimulation (≥5 Hz) exerts an excitatory effect (35). LF-rTMS is associated with minimal side effects, does not lead to drug dependence in adolescents, and effectively modulates the metabolic efficiency and cortical excitability of the dorsolateral prefrontal cortex, supplementary motor area, and the base of the frontal cortex, making it an acceptable initial treatment option for early-onset OCD (36). In our study, both 2-week and 4-week data revealed significant differences in treatment efficacy across groups. After 2 weeks of treatment, all groups demonstrated notable improvements in Y-BOCS and CGI-SI scores, indicating that early interventions effectively mitigated obsessive-compulsive symptoms. Specifically, the LF-rTMS group showed marked improvement in regulating cortical excitability, making it particularly beneficial for patients with heightened neural excitability. However, this effect may be more transient, as the modulation of cortical activity by LF-rTMS is temporary (22). On the other hand, the Fluoxetine group showed sustained improvement in mood regulation and control of compulsive behaviors, consistent with the prolonged neurochemical modulation provided by pharmacotherapy (12).

By the 4th week of treatment, the combined treatment group was associated with greater reductions in Y-BOCS and CGI-SI scores. Model-estimated marginal means (**Table 4**) and omnibus tests (**Table 5**) confirmed significant Time and Time×Group effects, indicating that symptom trajectories differed across treatment groups. These findings suggest a potential short-term advantage of combined treatment, although the observational design precludes causal inference. Moreover, the combined treatment group appeared to have more favorable changes in cognitive function and overall quality of life, which may support the possibility that integrating pharmacological treatment with neuromodulation could lead to better outcomes (36).

According to the result of this study, we can see that each treatment modality demonstrated unique strengths at different points in the treatment process. The LF-rTMS group was associated with early improvements in neuromodulation, making it suitable for patients with cortical hyperexcitability. The Fluoxetine group appeared to provide more sustained regulation of mood and compulsive behaviors, benefiting patients requiring longer pharmacological intervention. However, the combined treatment group showed more favorable short-term outcomes compared with the monotherapy groups, by integrating the neuromodulatory benefits of LF-rTMS with the prolonged effects of fluoxetine, which may contribute to more comprehensive symptom control and cognitive recovery. This suggests the potential value of multimodal interventions in managing early-onset OCD and indicates a need for confirmation in prospective studies (37).

The WCST is a key neuropsychological assessment used to evaluate cognitive flexibility, rule identification, and strategy adjustment (38). In our study, repeated-measures analyses demonstrated significant improvements across all treatment groups in WCST performance (**Figures 1A–E**). By week 4, the combination group was associated with the most favorable EMM values for correct responses and complete classification, and the largest reductions in perseverative and random errors. These results align with the omnibus tests (**Table 5**), which showed significant Time effects and Time×Group interactions for cognitive outcomes. Nevertheless, repeated administration of the WCST over a short interval may introduce practice effects, and part of the observed improvements could reflect test familiarity rather than purely treatment-induced cognitive change.

The reduction in perseverative errors across all groups, particularly in the combined therapy group, may reflect enhanced cognitive flexibility and improved error detection and correction abilities. LF-rTMS, as previously noted, can influence the excitability of the dorsal anterior cingulate cortex (39), a region linked to error monitoring and cognitive control. When combined with fluoxetine, which enhances serotonergic transmission and may improve neuroplasticity through increased BDNF expression (40, 41), the dual approach may help explain the observed improvements in cognitive function more effectively than either treatment alone. Moreover, the reduction in “random errors” was associated with improved attention and decision-making processes, which are often impaired in OCD patients. These findings are consistent with the possibility that LF-rTMS reduces the hyperactivity of OCD-related brain circuits, while fluoxetine stabilizes neurochemical imbalances (42). Together, these mechanisms may underlie the associations observed in this study, though causality cannot be inferred. Therefore, our study suggests the importance of a multimodal approach in treating adolescents with initial onset OCD. While both LF-rTMS and fluoxetine show significant individual benefits in improving cognitive function and reducing obsessive-compulsive symptoms, their combination appears to produce superior outcomes in terms of both cognitive enhancement and symptom relief (37).

In this study, qEEG was utilized to assess changes in brain activity across different frequency bands—α, β, θ, and δ—before and after 4 weeks of treatment. Under an eyes-closed resting-state protocol with standardized artifact handling (see Methods), qEEG provides indirect, non-invasive spectral indices of large-scale cortical rhythms in adolescents with initial onset OCD (43). The frequency of brain waves from EEG analysis typically ranges from approximately 0.1 to 100 Hz. Internationally, brain wave frequencies are generally categorized into four major bands: α, β, δ and θ (44). More specifically, α and β are considered fast wave bands, while δ and θ are slow wave bands. In our study, the combined treatment group exhibited higher α-band absolute power relative to the monotherapy groups; this pattern is consistent with altered cortical rhythm regulation but is non-specific and should not be equated with “relaxation” in the absence of corroborating physiological or behavioral measures. In the β band, regional differences were observed; in a resting-state paradigm such differences are also non-specific and may reflect multiple processes rather than discrete attentional states. The combined treatment was also associated with changes in θ and δ bands; given the indirect nature of spectral power, interpretation of these slow-wave alterations remains exploratory. Importantly, qEEG power cannot index neurotransmitter activity; mechanistic inferences (e.g., dopaminergic or serotonergic changes) cannot be drawn from resting-state spectra alone and would require multimodal confirmation. Taken together, the qEEG findings should be regarded as exploratory associations that align with the clinical patterns but remain preliminary given residual uncertainty in vigilance control, potential residual artifacts, and the short 4-week observation window (45, 46).

This single-center, retrospective study used preference-based treatment allocation rather than randomization, introducing selection bias and the possibility of residual confounding from measured and unmeasured factors; group sizes were unequal, which may have further affected comparability. The 4-week follow-up limits inference about durability of effects and is particularly insufficient for fluoxetine, which typically requires 8–12 weeks to exert robust anti-obsessional efficacy; thus, fluoxetine’s effect may be underestimated and direct comparisons with more rapidly acting LF-rTMS should be interpreted cautiously. We recalculated efficacy using a ≥30% Y-BOCS reduction to align with field standards; nevertheless, response estimates remain sensitive to the chosen cut-off and should be compared across studies with care. Repeated-measures models (LMM/GEE) were applied to account for within-subject correlation, but modest and imbalanced sample sizes may limit model stability and power for between-group contrasts. For WCST, repeated administration over 4 weeks may introduce practice effects in the absence of parallel forms. Although qEEG acquisition and artifact handling were standardized (eyes-closed rest; predefined rejection criteria), residual ocular/EMG contamination and vigilance variability cannot be fully excluded, and resting-state spectra provide non-specific indices that do not permit mechanistic inferences. Finally, generalizability beyond a single center is uncertain and these findings should be regarded as associative and hypothesis-generating rather than confirmatory; larger, prospective randomized controlled trials with longer follow-up are warranted.

## 5 Conclusion

This retrospective, non-randomized study found that combined fluoxetine and LF-rTMS treatment was associated with greater short-term improvements in symptom severity, cognitive function, and neural activity compared with monotherapy in adolescents with first-onset OCD. The combination also showed a favorable safety profile, with no notable increase in adverse effects relative to single treatments. These observational findings should be interpreted as preliminary and hypothesis-generating, as treatment allocation was preference-based, follow-up was limited to 4 weeks, and causal inference cannot be drawn. Nonetheless, the results suggest the potential value of multimodal strategies for enhancing symptomatic relief and cognitive outcomes, warranting confirmation in larger, prospective randomized controlled trials.

## Data Availability

The original contributions presented in the study are included in the article/**supplementary material**. Further inquiries can be directed to the corresponding author.
