# Supplementary material for: Short-term outcomes associated with fluoxetine, low-frequency rTMS, and their combination in first-onset adolescent OCD: a single-center retrospective cohort
Source: Front Psychiatry. 2025 Nov 5;16:1663611. doi: 10.3389/fpsyt.2025.1663611 (PMC12627030; doi:10.3389/fpsyt.2025.1663611)
Supplement: Supplementary file 1 [file DataSheet1.docx]

Supplementary materials 1.Yale-brown obsessive compulsive scale

Yale-brown obsessive compulsive scale（Y-BOCS）

1. How much time do obsessive thoughts occupy in your day?

Options:

- 0 hours, Score: 0

- 0-1 hour, Score: 1

- 1-3 hours, Score: 2

- 3-8 hours, Score: 3

- More than 8 hours, Score: 4

2. To what extent do obsessive thoughts affect your daily life or work?

Options:

- Not at all, Score: 0

- Mildly, Score: 1

- Moderately, Score: 2

- Severely, Score: 3

- Completely unable to work or engage in daily activities, Score: 4

3. How much distress do obsessive thoughts cause you when they occur?

Options:

- No distress, Score: 0

- Mild, Score: 1

- Moderate, Score: 2

- Severe, Score: 3

- Very severe, persistently distressing, Score: 4

4. How much effort do you exert to resist obsessive thoughts when they occur?

Options:

- Make every effort to resist, Score: 0

- Resist most obsessive thoughts, Score: 1

- Resist some obsessive thoughts, Score: 2

- Yield unwillingly, Score: 3

- Willingly do not resist, Score: 4

5. How well can you control your obsessive thoughts?

Options:

- Completely, Score: 0

- For the most part, Score: 1

- Usually, Score: 2

- With difficulty, Score: 3

- Not at all, Score: 4

6. How much time do compulsive behaviors occupy in your day?

Options:

- 0 hours, Score: 0

- 0-1 hour, Score: 1

- 1-3 hours, Score: 2

- 3-8 hours, Score: 3

- More than 8 hours, Score: 4

7. To what extent do compulsive behaviors affect your daily life or work?

Options:

- Not at all, Score: 0

- Mildly, Score: 1

- Moderately, Score: 2

- Severely, Score: 3

- Completely unable to work or engage in daily activities, Score: 4

8. How much distress do compulsive behaviors cause you when they occur?

Options:

- No distress, Score: 0

- Mild, Score: 1

- Moderate, Score: 2

- Severe, Score: 3

- Very severe, persistently distressing, Score: 4

9. How much effort do you exert to resist compulsive behaviors when they occur?

Options:

- Make every effort to resist, Score: 0

- Resist most compulsive behaviors, Score: 1

- Resist some compulsive behaviors, Score: 2

- Yield unwillingly, Score: 3

- Willingly do not resist, Score: 4

10. How well can you control your compulsive behaviors?

Options:

- Completely, Score: 0

- For the most part, Score: 1

- Usually, Score: 2

- With difficulty, Score: 3

- Not at all, Score: 4

Total Score and Assessment:

0-7: Based on the assessment, you are "Normal." We hope you continue to maintain a positive mood. (These test results are for reference only)

8-15: Based on the assessment, you have "Mild Obsessive-Compulsive Disorder." (These test results are for reference only)

16-23: Based on the assessment, you have "Moderate Obsessive-Compulsive Disorder." (These test results are for reference only)

24-31: Based on the assessment, you have "Severe Obsessive-Compulsive Disorder." (These test results are for reference only)

32-40: Based on the assessment, you have "Extreme Obsessive-Compulsive Disorder." (These test results are for reference only)

Supplementary materials 2.Treatment Emergent Symptom Scale

| Treatment Emergent Symptom Scale(TESS) | | | | | | | |
| --- | --- | --- | --- | --- | --- | --- | --- |
| Name |  | Gender |  | Age |  | Ward |  |
| Bed number |  | Appraiser |  |  |  |  |  |
|  | Severity | Measures | Severity | Measures | Severity | Measures | Severity |
| 1.Drowsiness |  |  |  |  |  |  |  |
| 2.Nausea |  |  |  |  |  |  |  |
| 3.Dizziness |  |  |  |  |  |  |  |
| 4.Elevation of blood pressure |  |  |  |  |  |  |  |
| 5.Toxic Confusional State |  |  |  |  |  |  |  |
| 6.Excitement or Agitation |  |  |  |  |  |  |  |
| 7.Emotional Depression |  |  |  |  |  |  |  |
| 8.Increased Activity |  |  |  |  |  |  |  |
| 9.Decreased Activity |  |  |  |  |  |  |  |
| 10.Insomnia |  |  |  |  |  |  |  |
| 11.Abnormal Blood Count |  |  |  |  |  |  |  |
| 12.Abnormal Liver Function |  |  |  |  |  |  |  |
| 13.Abnormal Urine |  |  |  |  |  |  |  |
| 14.Muscle Rigidity |  |  |  |  |  |  |  |
| 15.Tremor |  |  |  |  |  |  |  |
| 16.Torsional Movements |  |  |  |  |  |  |  |
| 17.Akathisia |  |  |  |  |  |  |  |
| 18.Dry Mouth |  |  |  |  |  |  |  |
| 19.Nasal Congestion |  |  |  |  |  |  |  |
| 20.Blurred Vision |  |  |  |  |  |  |  |
| 21.Constipation |  |  |  |  |  |  |  |
| 22.Excessive Salivation |  |  |  |  |  |  |  |
| 23.Sweating |  |  |  |  |  |  |  |
| 24.Diarrhea |  |  |  |  |  |  |  |
| 25.Hypotension |  |  |  |  |  |  |  |
| 26.Tachycardia |  |  |  |  |  |  |  |
| 27.EKG Abnormalities |  |  |  |  |  |  |  |
| 28.Skin Symptoms |  |  |  |  |  |  |  |
| 29.Weight Gain |  |  |  |  |  |  |  |
| 30.Weight Loss |  |  |  |  |  |  |  |
| 31.Decreased Appetite or Anorexia |  |  |  |  |  |  |  |
| 32.Headache |  |  |  |  |  |  |  |
| 33.Tardive Dyskinesia |  |  |  |  |  |  |  |
| 34.Others |  |  |  |  |  |  |  |
| Severity: 0 none; 1 mild or very slight; 2 moderate; 3 severe; 4 very severe | | | | | | | |
| Measures: 0 none; 1 strengthen observation; 2 administer appropriate antagonists; 3 reduce dosage; 4 reduce dosage and administer appropriate antagonists; 5 suspend treatment 6 terminate treatment | | | | | | | |

Supplementary materials 3.Clinical Global Impressions

Clinical Global Impressions (CGI)

1. Severity of Illness (SI)

Considering your total clinical experience with this particular population, how mentally ill is the patient at this time?

Normal, not at all ill, Score: 1

Borderline mentally ill, Score: 2

Mildly ill, Score: 3

Moderately ill, Score: 4

Markedly ill, Score: 5

Severely ill, Score: 6

Among the most extremely ill patients, Score: 7

2. Global Improvement (GI)

Rate total improvement whether or not in your judgment it is due entirely to drugtreatment. Compared to his/her condition at admission to the project, how much has he/she changed?

Very much improved, Score: 1

Much improved, Score: 2

Minimally improved, Score: 3

No change, Score: 4

Minimally worse, Score: 5

Much worse, Score: 6

Very much worse, Score: 7

3. Efficacy Index (EI)

Rate this item on the basis of drug effect only. Select the terms that best

describe the degrees of therapeutic effect and side effects and make a mark in the box where the two items intersect.

| Therapeutic Effect | Side Effects | | | |
| --- | --- | --- | --- | --- |
|  | None | Do not significantly interfere with patient's functioning | Significantly interfere with patient's functioning | Outweigh therapeutic effect |
|  | 1 | 2 | 3 | 4 |
| 4. Marked: Vast improvement. Complete or nearly complete remission of all symptoms. | 4 | 2 | 1.33 | 1 |
| 3. Moderate: Decided improvement. Partial remission of symptoms. | 3 | 1.5 | 1 | 0.75 |
| 2. Minimal: Slight improvement that doesn't alter status of care of patient. | 2 | 1 | 0.67 | 0.5 |
| 1. Unchanged or Worse | 1 | 0.5 | 0.33 | 0.25 |
